# Geographic Variation in Sexual Attraction of *Spodoptera frugiperda* Corn- and Rice-Strain Males to Pheromone Lures

**DOI:** 10.1371/journal.pone.0089255

**Published:** 2014-02-19

**Authors:** Melanie Unbehend, Sabine Hänniger, Gissella M. Vásquez, María Laura Juárez, Dominic Reisig, Jeremy N. McNeil, Robert L. Meagher, David A. Jenkins, David G. Heckel, Astrid T. Groot

**Affiliations:** 1 Department of Entomology, Max Planck Institute for Chemical Ecology (MPICE), Jena, Germany; 2 Department of Entomology, North Carolina State University, Raleigh, North Carolina, United States of America; 3 Sección Zoología Agrícola, Estación Experimental Agroindustrial Obispo Colombres, Las Talitas, Tucumán, Argentina; 4 Department of Entomology, North Carolina State University, Plymouth, North Carolina, United States of America; 5 Department of Biology, University of Western Ontario, London, Ontario, Canada; 6 Insect Behavior and Biocontrol Research Unit, United States Department of Agriculture, Gainesville, Florida, United States of America; 7 Tropical Crops and Germplasm Research, United States Department of Agriculture, Mayaguez, Puerto Rico; 8 Institute for Biodiversity and Ecosystem Dynamics, University of Amsterdam, Amsterdam, the Netherlands; Plant and Food Research, New Zealand

## Abstract

The corn- and rice-strains of *Spodoptera frugiperda* exhibit several genetic and behavioral differences and appear to be undergoing ecological speciation in sympatry. Previous studies reported conflicting results when investigating male attraction to pheromone lures in different regions, but this could have been due to inter-strain and/or geographic differences. Therefore, we investigated whether corn- and rice-strain males differed in their response to different synthetic pheromone blends in different regions in North America, the Caribbean and South America. All trapped males were strain-typed by two strain-specific mitochondrial DNA markers. In the first experiment, we found a nearly similar response of corn- and rice-strain males to two different 4-component blends, resembling the corn- and rice-strain female blend we previously described from females in Florida. This response showed some geographic variation in fields in Canada, North Carolina, Florida, Puerto Rico, and South America (Peru, Argentina). In dose-response experiments with the critical secondary sex pheromone component (Z)-7-dodecenyl acetate (Z7-12:OAc), we found some strain-specific differences in male attraction. While the response to Z7-12:OAc varied geographically in the corn-strain, rice-strain males showed almost no variation. We also found that the minor compound (Z)-11-hexadecenyl acetate (Z11-16:OAc) did not increase attraction of both strains in Florida and of corn-strain males in Peru. In a fourth experiment, where we added the stereo-isomer of the critical sex pheromone component, (E)-7-dodecenyl acetate, to the major pheromone component (Z)-9-tetradecenyl acetate (Z9-14:OAc), we found that this compound was attractive to males in North Carolina, but not to males in Peru. Overall, our results suggest that both strains show rather geographic than strain-specific differences in their response to pheromone lures, and that regional sexual communication differences might cause geographic differentiation between populations.

## Introduction

Geographic variation in the sexual communication signals of animals is a widespread phenomenon, being reported in frogs [1]–[3], birds [4]–[6], fish [7] and insects [8]–[10]. This variation can be the result of isolation by distance, with a positive correlation between genetic dissimilarity and geographic distance [11]–[13], but this is not always the case [14]–[16]. Furthermore, mating signals can be influenced by environmental factors such as temperature [17]–[20], humidity [21]–[23], photoperiod length [24], [25], host plant volatiles [26], [27] or interspecific olfactory cues [28] that vary geographically.

Geographic variation in sexual communication systems has been reported in several lepidopteran species [29]–[34] and is of interest because changes in the sex pheromone signal and/or response to sex pheromones could result in reproductive isolation and subsequently may lead to speciation [35]–[38]. Furthermore, geographically varying sexual communication is of interest for pest management, as many lepidopteran insects are pest species which are commonly monitored, disrupted or killed via pheromone-mediated methods [39]–[41].

The fall armyworm, *Spodoptera frugiperda* (J. E. Smith) (Lepidoptera: Noctuidae), consists of two genetically and behaviorally distinct strains, the corn- and rice-strain, occurring sympatrically throughout North- and South America [42]. Both strains appear to be undergoing ecological speciation in sympatry and reveal several possible prezygotic isolation barriers [43]. These include differential host plant choice [42], [44]–[47], strain-specific mating times in the scotophase [48], [49], as well as differences in the female sex pheromone composition [50]–[52]. Among these prezygotic mating barriers, the strain-specific timing of reproduction seems to be the most important one that differentiates both strains, as host plant preference is not as clear cut as previously thought [43], [46], [53], [54], and no strain-specific mating based on sex pheromone differences could be shown so far [48], [52], [55]. Additionally, a postzygotic mating barrier, i.e. reduced fertility of RC (rice-strain ♀ x corn-strain ♂) hybrid females, contributes to the divergence of the two strains and separates them in nature [43]. Besides being an excellent model to study the evolution of reproductive isolation [43], *S. frugiperda* is a serious pest species that feeds on a large variety of agricultural crops [56], and can cause annual damages of up to ∼300 million dollars in the United States [57].

The sex pheromone of *S. frugiperda* was identified by Tumlinson et al. [58] to consists of (Z)-9-tetradecenyl acetate (Z9-14:OAc) as the major sex pheromone component, and (Z)-7-dodecenyl acetate (Z7-12:OAc) as critical secondary sex pheromone component. A number of other minor compounds like (Z)-11-hexadecenyl acetate (Z11-16:OAc) and (Z)-9-dodecenyl acetate (Z9-12:OAc) have also been identified from the gland [50], [51], [58], but with unclear behavioral function so far [52]. Analysis of sex pheromone gland extracts from females collected in Florida showed that corn-strain females contained significantly lower relative amounts of Z7-12:OAc and Z9-12:OAc than rice-strain females [50], [52]. However, different male trapping experiments conducted in Louisiana and Florida showed no consistent attraction of males to females of their own strain [48], [52], [55], which suggests that differences in the female pheromone are not sufficient to cause assortative mating in the field.

There is evidence that there are geographic differences in this species in the female sex pheromone blend [50], [51], [58], [59], as well as in the male response [52], [58]–[63]. For example, while females from Brazil [59] produce (E)-7-dodecenyl acetate (E7-12:OAc), those from Florida, Louisiana or French Guyana do not [50], [51], [64]. In addition, studies on females originating from Florida and Louisiana provide evidence of geographic variation in the production of sex pheromone by females of both strains [50]–[52]. Numerous studies have shown that the number of males caught varies with the pheromone blend used. For example, while the minor compound Z11-16:OAc did not affect male attraction in Florida and Brazil [52], [58], [59], it did marginally increase capture rates in Costa Rica [61], and addition of Z11-16:OAc and Z9-12:OAc to binary blends (of Z9-14:OAc and Z7-12:OAc) almost doubled the attraction of males in Pennsylvania [62]. However, most of these studies did not determine the strain identity of the males captured. Consequently, the variation in male attraction observed in these studies could either be due to strain-specific and/or due to geographic differences.

To disentangle strain-specific variation from geographic variation in male response, we investigated the response of corn- and rice-strain males to different synthetic pheromone blends in six different countries in North America, the Caribbean and South America. We tested (A) two synthetic 4-component blends (Blend 1 and 2) in different fields in Canada, North Carolina, Florida, Puerto Rico, Peru and Argentina; (B) different doses of Z7-12:OAc in Florida, Puerto Rico and Peru; (C) different doses of Z11-16:OAc in Florida and Peru; and (D) different doses of E7-12:OAc and Z7-12:OAc in Peru and North Carolina. We found that corn- and rice-strain males showed some variation in their response to different synthetic pheromone blends in different geographic regions. Overall, our results suggest that there is less strain-specific than geographic variation in male response and that regional sexual communication differences might cause geographic differentiation between populations.

## Materials and Methods

### Ethics Statement

The examined species is neither endangered nor protected. Trapping experiments in Canada, North Carolina and Peru were conducted at experimental research stations of the University of Western Ontario, the North Carolina State University, and the Universidad Nacional Agraria La Molina, with permission of the local university members/experimenters (Table 1). Trapping experiments in Florida were conducted at a) the Everglades Research and Education Center of the University of Florida in Belle Glade with permission of Gregg S. Nuessly, b) a private field in Hague with permission obtained by RLM, and c) private fields at the Graham Farm in Moore Haven with permission of the farm manager Tommy Toms (Table 1). Field experiments in Puerto Rico were conducted at field sites of the seed companies Monsanto and 3^rd^ Millenium Genetics with permission of the company employees Wilson Rivera González and Jose Santiago, respectively. In Argentina, experiments were conducted in a private field with permission obtained by MLJ (Table 1).

**Table 1:** *Spodoptera frugiperda* male trapping experiments conducted in North America, the Caribbean and South America.

COUNTRY	LOCATION & COORDINATES		FIELD	EXPERIMENT^1^	EXPERIMENTER	DATE
**Canada**	Ontario	+43° 4′ 26.08′′, −81° 20′ 21.81′′	Corn	A	JMN	Sep. 2011
**North Carolina**	Plymouth	+35°50′ 46.19′′, −76° 39′ 46.24′′	Soybean (Replicate 1)	A, D	DR	Sep. 2011
		+35°51′ 01.93′′, −76° 39′ 11.28′′	Cotton; Grass (Rep. 2)			
		+35°51′ 48.80′′, −76° 39′ 33.61′′	Soybean; Corn (Rep. 3)			
**Florida** 2	Belle Glade	+26° 40′ 7.20′′, −80° 37′ 57.63′′	Corn A	A, B	MU, SH	April–May 2010
	Hague	+29° 47′ 7.40′′, −82° 25′ 3.66′′	Corn B	C	RLM	Sept. 2011
	Moore Haven	+26° 53′ 3.04′′, −81° 7′ 21.17′′	Grass	A, B, C	MU, SH	April–May 2010
**Puerto Rico**	Santa Isabel	+17° 59′ 0.93′′, −66° 23′ 29.88′′	Corn A	A	MU, SH,	April 2010
		+17°57′ 30.65′′, −66° 23′ 32.43′′	Corn B	A, B	ATG, DAJ	April 2010
**Peru**	Lima	−12° 4′ 51.56′′, −76° 57′ 9.14′′	Corn	A, B, C, D	GMV	May–July 2011
**Argentina**	El Molino	−27° 20′ 11.1′′, −65° 41′ 25.8′′	Corn	A	MLJ	Dec. 2010–Jan. 2011

1 Experiments: A) Test of two 4-component blends (Blend 1 and Blend 2), B) Z7-12:OAc dose-response, C) Z11-16:OAc dose-response, D) Importance of E7-12:OAc.

2 Data adapted from [52].

### Male Trapping Experiments

To test whether a certain synthetic pheromone blend is equally attractive for corn- and rice-strain males in different geographic regions, four different trapping experiments were conducted in six different regions in North America, the Caribbean and South America (Table 1). For experiment (A), we prepared two synthetic 4-component blends (Blend 1 and 2) based on strain-specific pheromone differences found in laboratory females from Florida by Groot et al. [50]. Both blends consisted of 100% Z9-14:OAc, but with different percentages of Z11-16:OAc, Z7-12:OAc and Z9-12:OAc (Table 2), as described by Unbehend et al. [52]. Both blends were tested in Canada, the United States (North Carolina and Florida), Puerto Rico, Peru and Argentina (Table 1).

**Table 2:** Composition of pheromone lures used to attract *Spodoptera frugiperda* males in the field.

Experiment and Lures^1^			Z9-14:OAc	Z11-16:OAc	Z7-12:OAc	E7-12:OAc	Z9-12:OAc
**A**	Blend 1 (corn-strain blend)^2^		100%	13%	2%	−	1%
	Blend 2 (rice-strain blend)^2^		100%	8%	4%	−	2%
	Blank (Hexane)		−	−	−	−	−
**B**	0%	Z7-12:OAc	100%	−	−	−	−
	2%		100%	−	2%	−	−
	4%		100%	−	4%	−	−
	10%		100%	−	10%	−	−
**C**	0%	Z11-16:OAc	100%	−	2%	−	−
	8%		100%	8%	2%	−	−
	13%		100%	13%	2%	−	−
	18%		100%	18%	2%	−	−
**D**	2%	Z7-12:OAc	100%	−	2%	−	−
	2%	E7-12:OAc	100%	−	−	2%	−
	1+1%	Z/E7-12:OAc	100%	−	1%	1%	−
	2+2%	Z/E7-12:OAc	100%	−	2%	2%	−

1 Compound concentrations were as follows: 100% = 300 µg, 18% = 54 µg, 13% = 39 µg, 10% = 30 µg, 8% = 24 µg, 4% = 12 µg, 2% = 6 µg, 1% = 3 µg.

2 Blend 1 and Blend 2 were based on the pheromone gland composition of Florida corn- and rice-strain females, respectively [52].

To evaluate the relative importance of Z7-12:OAc for male attraction in Florida, Puerto Rico and Peru (experiment B), different percentages of Z7-12:OAc (0%, 2%, 4%, 10%) were added to the major sex pheromone component Z9-14:OAc alone (Table 2). The percentages used were chosen to examine whether Z7-12:OAc is necessary for male attraction in all regions and fields (0%, lures baited only with Z9-14:OAc), to test whether males can distinguish between 2% and 4% Z7-12:OAc, which is the difference that we found between corn- and rice-strain females [50], and to investigate a possible repellent effect of high dosages of Z7-12:OAc (10%).

To assess whether Z11-16:OAc would affect male attraction, we conducted experiment (C), in which different amounts of Z11-16:OAc (0%, 8%, 13%, 18%) were added to a “minimal blend”, consisting of 100% Z9-14:OAc and 2% Z7-12:OAc (Table 2). The “minimal blend” (0% Z11-16:OAc) was used as control, while 8% and 13% Z11-16:OAc reflect the percentages found in rice- and corn-strain females from Florida, respectively [50]. To test possible repellent effects, 18% was used as the highest concentration. Experiment C was conducted in Florida and Peru.

To test the importance of the isomers Z7-12:OAc and E7-12:OAc in North and South America (experiment D), we added different doses of E7-12:OAc and Z7-12:OAc (0%, 1%, 2%) to 100% Z9-14:OAc (Table 2). The “minimal blend” (2% Z7-12:OAc +100% Z9-14:OAc) was used as control, and as an equivalent we prepared an E-blend with 2% E7-12:OAc and 100% Z9-14:OAc. To investigate a possible interaction effect of both isomers together, 1% as well as 2% of E- and Z7-12:OAc were added to 100% Z9-14:OAc. The fourth experiment (Exp. D) was carried out in North Carolina and Peru.

We were not able to conduct all four experiments in all countries, due to technical limitations (i.e. limited time availability of collaborators, limited access to infested field sites and variability of moth population densities). All data from trapping experiments in Florida were published previously [52] and were included in this study for comparison. In all experiments, the synthetic pheromone lures were placed in plastic green-yellow-white Unitraps (Pherobank, Wageningen, the Netherlands), which contained a Vaportape II insecticide strip (Hercon Environmental, Emigsville, PA, USA) to kill the males captured. At each site, traps were hung just above the crop canopy (1–2 m above the ground depending on crop phenology), spaced 15 m apart and at least 15 m from the edge of the field using a complete randomized block design. There were three replicates per treatment per field (n = 3), except for experiments conducted in North Carolina, where each replicate was conducted in a different field (Table 1). Traps were rotated and emptied three or four times, depending on the number of treatments (Exp. A: n = 3; Exp. B-D: n = 4), and traps were rotated every 1–6 days, depending on the population density in the field. The males captured were stored at −20°C until strain-identification in the laboratory (see below).

### Preparation of Pheromone Lures

All pheromone compounds used to prepare lures were bought from Pherobank (Wageningen, the Netherlands), and had a purity of ≥99%. Red rubber septa (Thomas Scientific, Swedesboro, NJ, USA) were soaked in hexane for 24 hours and air dried before they were loaded with 100 µl hexane containing 300 µg of the major pheromone component, Z9-14:OAc, plus different amounts (relative to 300 µg Z9-14:OAc) of the minor compounds Z11-16:OAc, Z7-12:OAc, Z9-12:OAc, and E7-12:OAc (Table 2). To avoid variable loading of our multi-component lures, all components of one specific lure were mixed together in hexane, formulated into one blend, and checked on the gas chromatograph before they were loaded onto a septum. All lures within one experiment were prepared using one master solution to avoid variation between replicates. The loaded relative amounts of each minor pheromone component reflected the relative amounts present in the female pheromone gland [50]. To avoid variation in attraction due to differential amounts of the major component, the amount of Z9-14:OAc was set to 100% in all blends (Table 2), similar to studies conducted by Batista-Pereira et al. [59], and Groot et al. [65], [66]. Therefore, the sum of all components in our multi-component blends was always larger than 100%, and the total amount of pheromone per lure ranged from 300 µg up to 360 µg (Table 2). All prepared lures were stored in glass vials at −20°C until used 1–3 months later in the field. Each lure was only used once within one experiment (for ∼1–3 weeks), and we did not observe a consistent decrease in lure-effectiveness at the end of an experiment (Figure S1). However, we cannot completely exclude a decrease in emission rates over time.

### Chemical Analysis

The purity and composition of the prepared pheromone solutions were verified by gas chromatography (GC) analysis, using a HP7890 gas chromatograph with a 7683 automatic injector. A 2 µl aliquot of each pheromone solution used for the preparation of the pheromone lures (Table 2) was injected into a splitless inlet attached to a polar capillary column (DB-WAXetr; 30 m×0.25 mm×0.5 µm) and a flame-ionization detector (FID). The GC program ran from 60°C, with a 2 min hold, to 180°C at 30°C/min, 230°C at 5°C/min and to 245°C at 20°C/min, followed by a 15 min hold at 245°C to clean the column for the next sample. The FID detector was held at 250°C.

### Strain Identification

The strain identity of all trapped males was determined via two strain-specific markers, i.e. MspI- and SacI-digest of the mitochondrial COI gene, which are known to be diagnostic for strain-identification of the two fall armyworm strains in North and South America [46], [53], [67]. DNA of all males captured was extracted as described by Unbehend et al. [52] using CTAB (Cetyltrimethylammonium bromide) and isopropanol for DNA precipitation. The extracted DNA was tested at MPICE for strain-specific polymorphisms at the mitochondrial COI gene by amplification and strain-specific digestion [46], [52]. The amplified part of the COI gene was digested with MspI as well as SacI and analyzed electrophoretically on a 1% agarose gel [52]. MspI digestion detected corn-strain individuals, whereas SacI digestion proved rice-strain identity [46].

### Statistical Analysis

Data of each field site of one experiment (Exp. A – D) were analyzed separately, using a generalized linear model (GLM) with a Poisson distribution, or a quasi-Poisson distribution if the residual deviance of the data was larger than the residual degrees of freedom (over-dispersion), using the R software 2.11.1 [68]. To assess whether there was any effect of geographic location, field crop, and/or any strain-specific effect that influenced male attraction, data of experiment A and B were additionally analyzed with a multivariate analysis of variance (MANOVA) and a Wilks Lambda test. Untransformed data were used for all GLM analyses, while data were square root transformed for the MANOVA. Treatments that did not catch any moths within any rotation in any of the three biological replicates per field were excluded from the statistical analysis (e.g. blanks in Experiment A). Whenever a certain blend attracted one or more males in at least one trap within one biological replicate, possible zero catches of the other biological replicates were included in the analysis. In all graphs, we averaged the number of males of all rotations of one treatment, calculated one percentage value for each of the 3 biological replicates, and plotted the mean percentage of males per trap (i.e. the average of the 3 biological replicates). In the statistical analysis, only raw data (no means or percentages) were used.

## Results

**Figure 1:**
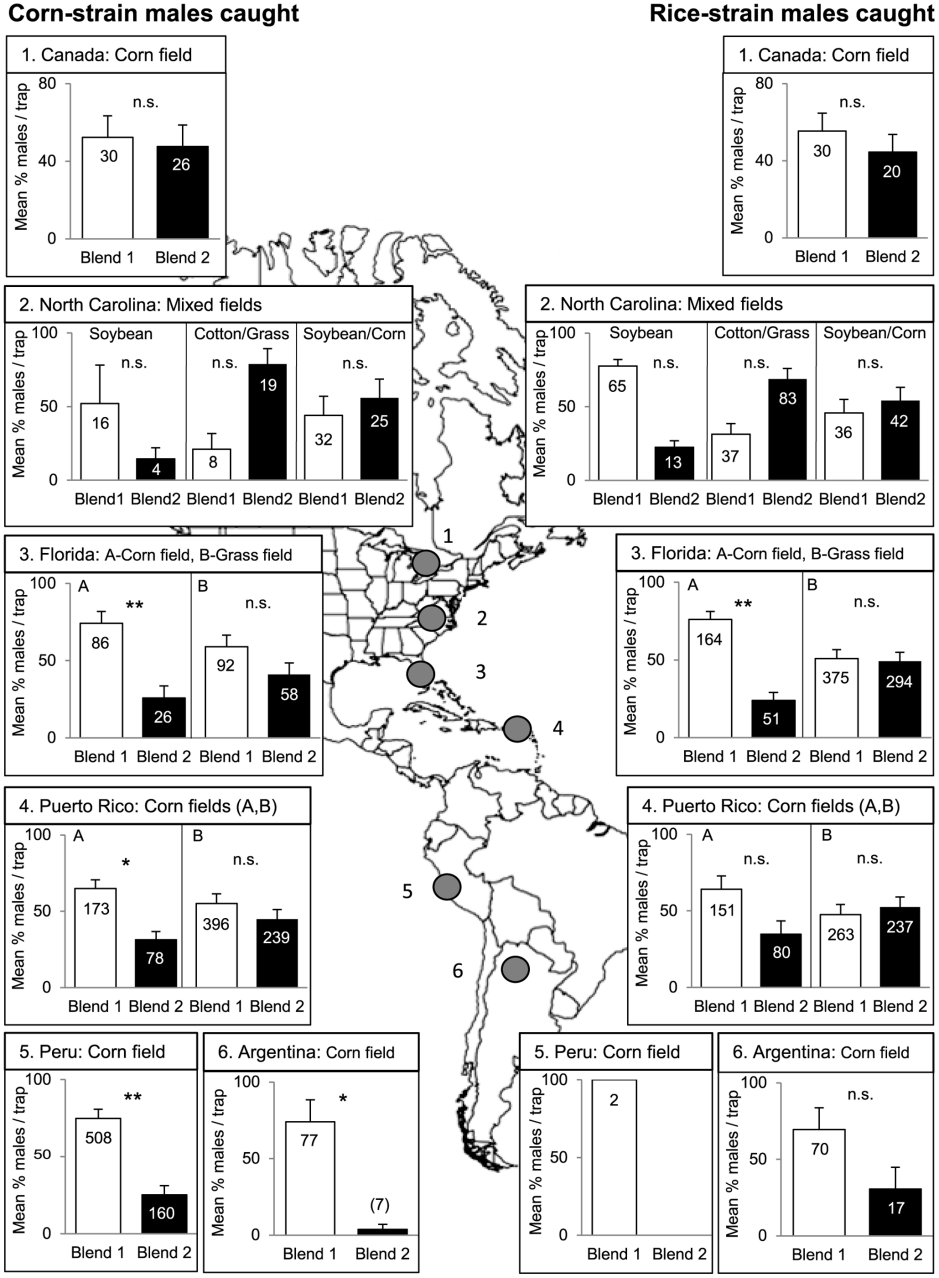
Attraction of *Spodoptera frugiperda* corn- and rice-strain males to two 4-component blends in different regions. Bars show the mean percentage of males caught per trap (Blend 1∶100% Z9-14:OAc +13% Z11-16:OAc +2% Z7-12:OAc +1% Z9-12:OAc; Blend 2∶100% Z9-14:OAc +8% Z11-16:OAc +4% Z7-12:OAc +2% Z9-12:OAc) and per biological replicate. There were three biological replicates per field (n = 3), except for all fields in North Carolina (n = 1) and for rice-strain males in Peru (n = 1), where only one replicate caught males. The standard errors in all fields in North Carolina show the variation between rotations in one replicate (n = 3), while all other error bars show the variation between biological replicates (n = 3). Numbers in brackets/bars represent the total number of males caught,* = *P*<0.05,** = *P*<0.01, n.s. = not significant. Data from Florida are adapted from [52].

Overall, the field tests showed that *S. frugiperda* males of both strains exhibited some geographic variation in their attraction to two different synthetic 4-component-blends (Blend 1 and Blend 2) in North America, the Caribbean and South America (Figure 1). We found a significant effect of geographic region, field crop, as well as an interaction effect between geographic region x strain (*P*<0.001, Table 3). Corn-strain males showed a significantly higher attraction to Blend 1 than to Blend 2 in corn fields in Florida, Puerto Rico (field A), Peru and Argentina, but did not show a preference for any of the two blends in corn fields in Canada and Puerto Rico (field B), the mixed habitats in North Carolina or a grass field in Florida (Figure 1). Rice-strain males were equally attracted to Blend 1 and Blend 2 in all cases, with only one exception in a corn field in Florida, where Blend 1 was more attractive than Blend 2. Both strains only differentiated between the two blends when the experiment was conducted in a corn field, where males of both strains were more attracted to Blend 1 than to Blend 2 (Figure 1). Control traps baited with hexane were usually empty in all fields (data not shown), but did trap some males in Argentina (n = 2) and in Puerto Rico (n = 19 in field A, n = 2 in field B). Interestingly, 18 out of the 19 males found in control traps in corn field A in Puerto Rico were caught during the first trap rotation at a time where male density was extremely high (over 50% of all males caught in this experiment were caught at the date of the first rotation, Figure S1).

**Table 3:** Test statistics on the *Spodoptera frugiperda* male trap catches of different experiments.

	EXPERIMENT A			EXPERIMENT B		
	Wilks	F	P	Wilks	F	P
Country	0.448	12.152	**<0.001**	0.196	26.711	**<0.001**
Strain	0.975	1.551	0.216	0.697	9.245	**<0.001**
Field	0.619	8.345	**<0.001**	0.907	2.181	0.078
Country:Strain	0.471	11.245	**<0.001**	0.425	11.333	**<0.001**
Strain:Field	0.899	1.680	0.104	0.787	5.743	**<0.001**

**Figure 2:**
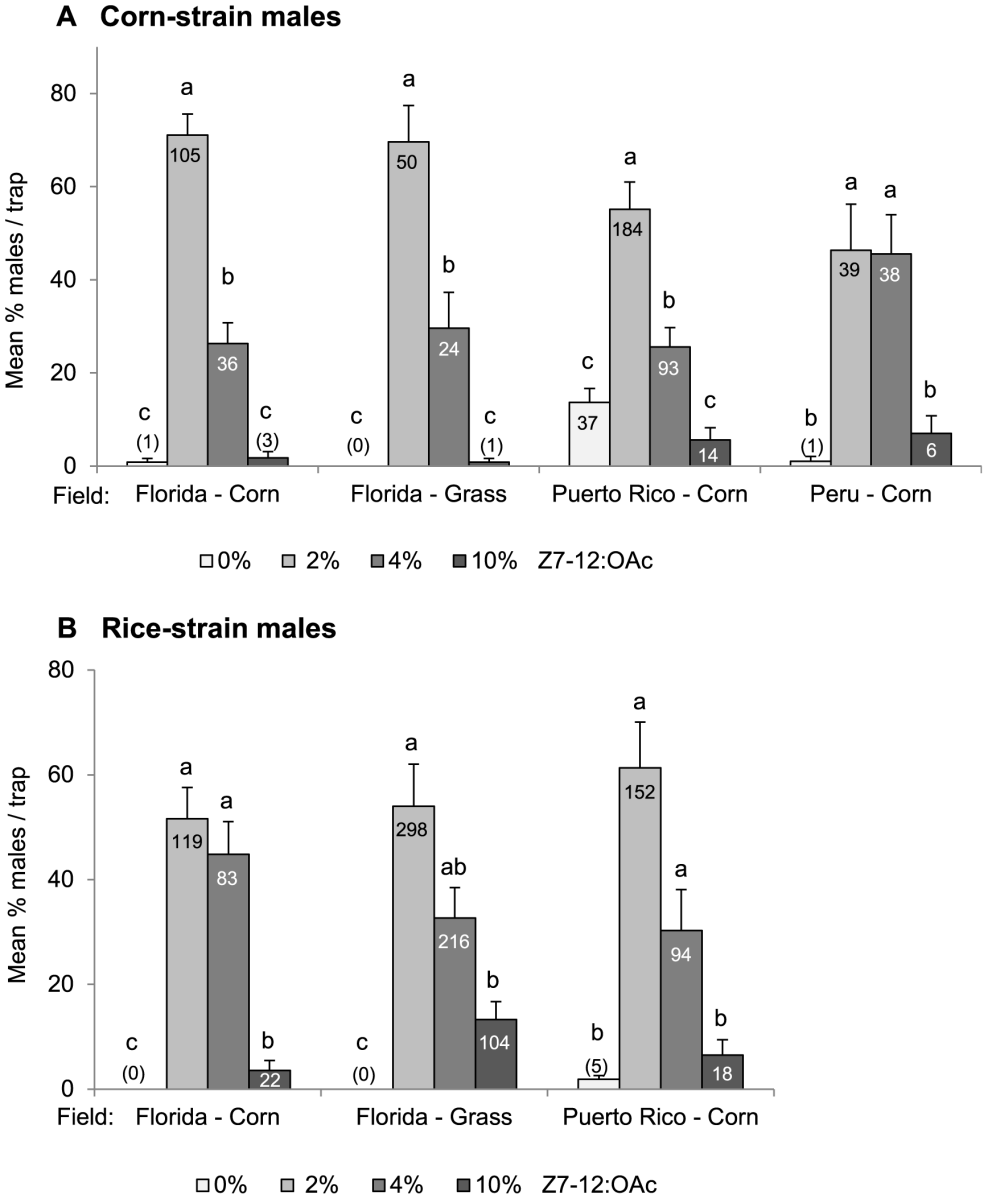
Attraction of corn-strain (A) and rice-strain (B) males to different doses of Z7-12:OAc. Bars represent the mean percentage of males caught per trap (0%, 2%, 4%, or 10% Z7-12:OAc +100% Z9-14:OAc) and per biological replicate (n = 3). Different letters above the bars indicate significant differences. Error bars show the variation between biological replicates (n = 3). Numbers in brackets/bars represent the total number of males caught. Data from Florida are adapted from [52].

Experiments A (Test of two 4-component blends: Blend 1 and Blend 2) and B (Z7-12:OAc dose-response experiment) were analyzed individually using square root transformed data in a MANOVA and a Wilks’ Lambda test. Bold P-values show a significant effect of geographic region, strain-identity of males, and/or the field crop, influencing the attraction of fall armyworm males to synthetic pheromone blends. Mean values and standard errors are shown in Figure 1 (Exp. A) and Figure 2 (Exp. B).

In experiment B, the Z7-12:OAc dose-response experiment, we found a significant effect of geographic region and strain, as well as an interaction effect between geographic region x strain, and field crop x strain (*P*<0.001; Table 3). Interestingly, corn-strain males exhibited a greater differentiation in their response to Z7-12:OAc than rice-strain males (Figure 2), i.e. the highest number of corn-strain males was captured with lures containing 2% Z7-12:OAc, which was significantly different from all other ratios at three field sites in Florida and Puerto Rico. In Peru, lures with 2% and 4% Z7-12:OAc captured equal numbers of corn-strain males (Figure 2A). In Puerto Rico, lures containing no Z7-12:OAc, which is the critical secondary sex pheromone component of this species, attracted 37 corn-strain males. In contrast to corn-strain males, rice-strain males showed a similar response to different concentrations of Z7-12:OAc in all regions tested, being equally attracted to blends containing 2% or 4% Z7-12:OAc, and showing some level of response to lures with 10% Z7-12:OAc (Figure 2B). No data of rice-strain males could be gathered in Peru, as only corn-strain males were found in this field (Figure 2A).

**Figure 3:**
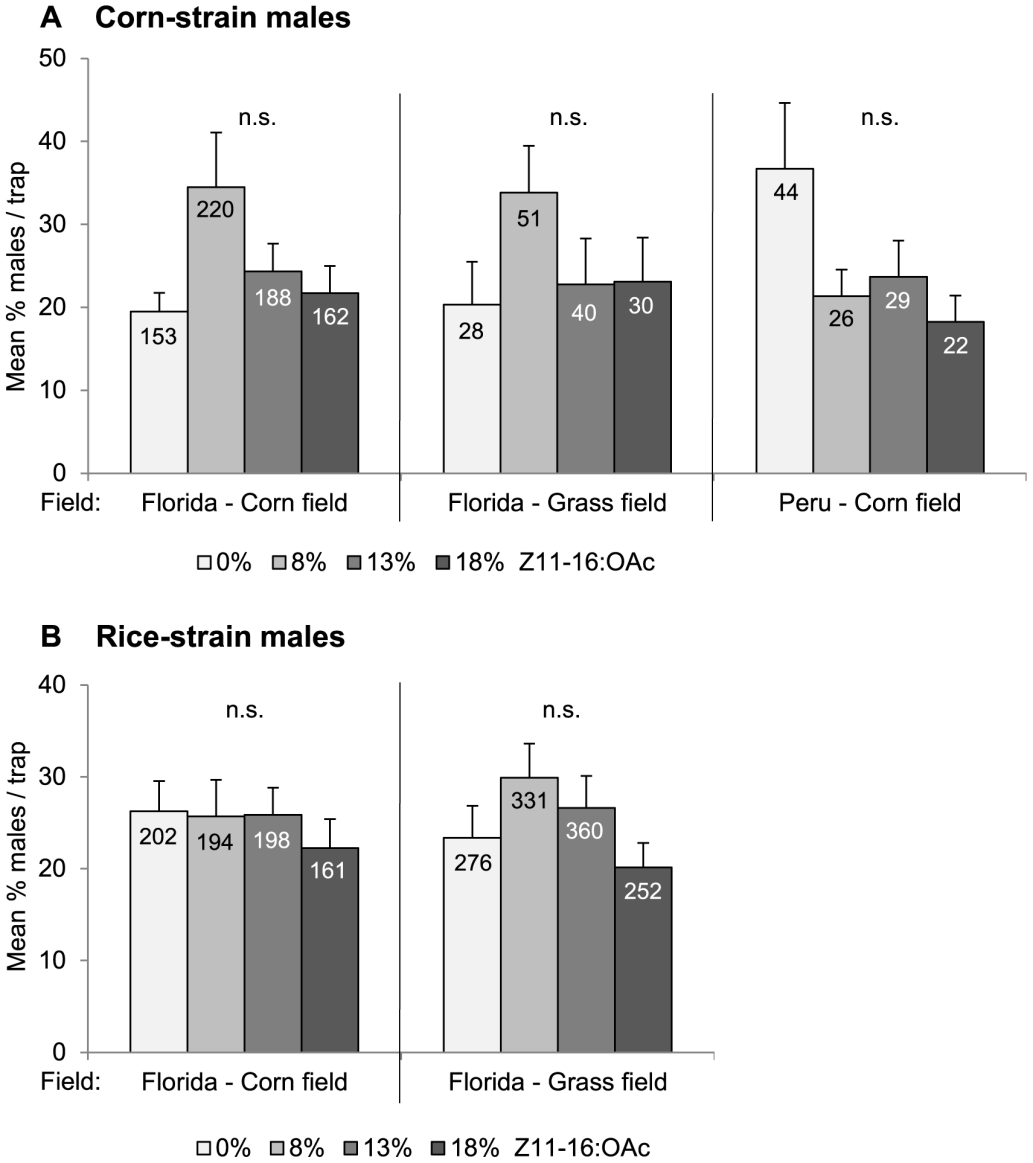
Attraction of corn-strain (A) and rice-strain (B) males to different doses of Z11-16:OAc. Bars represent the mean percentage of males caught per trap (0%, 8%, 13%, or 18% Z11-16:OAc +100% Z9-14:OAc +2% Z7-12:OAc) and per biological replicate (n = 3). Error bars show the variation between biological replicates (n = 3). Numbers in the bars represent the total number of males caught. n.s. = not significant. Data from Florida are adapted from [52].

Testing different doses of Z11-16:OAc (experiment C) added to the minimal blend (i.e. 100% Z9-14:OAc and 2% Z7-12:OAc), revealed that corn-strain males were similarly attracted to binary blends as to three-component blends containing 8%, 13% or 18% Z11-16:OAc (Figure 3A). This was true in Peru and in Florida. Similarly, addition of Z11-16:OAc did not influence the attraction of rice-strain males in Florida (Figure 3B). In Peru, no rice-strain males were caught in the corn field.

**Figure 4:**
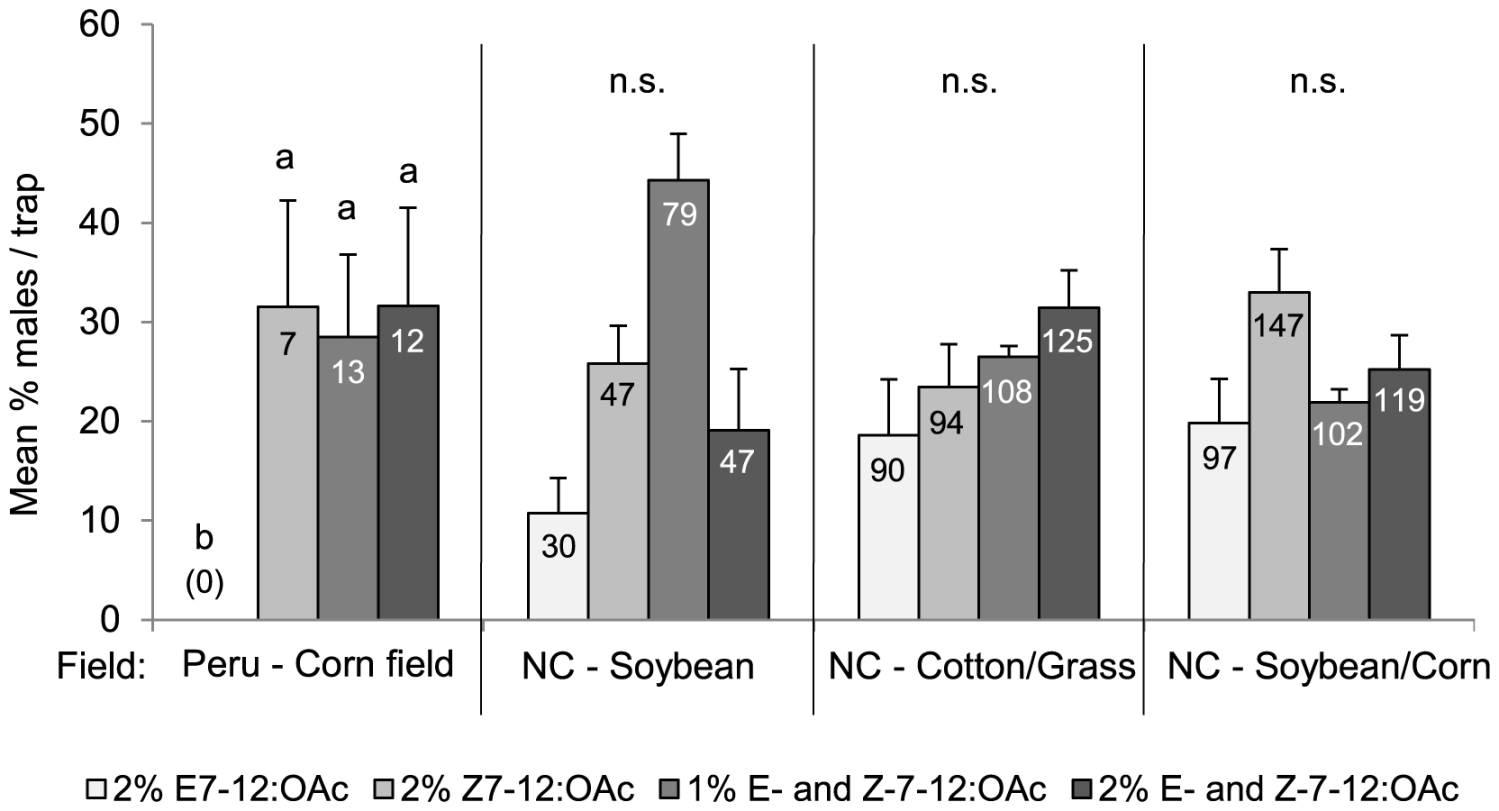
Attraction of *Spodoptera frugiperda* males to different doses of E7-12:OAc and Z7-12:OAc in different fields. Bars show the mean percentage of males caught per trap (100% Z9-14:OAc+a: 2% E7-12:OAc, b: 2% Z7-12:OAc, c: 1% E- and Z-7-12:OAc, d: 2% E- and Z-7-12:OAc) and per biological replicate. The standard errors in all fields in North Carolina (NC) show the variation between rotations in one replicate (n = 3), while error bars in Peru show the variation between biological replicates (n = 3). Males from Peru are corn-strain males, while males from NC could not be strain-typed but probably belonged to both strains. Numbers in brackets/bars represent the total number of males caught. Different letters above the bars indicate significant differences. n.s. = not significant.

In experiment D, when testing different doses (0%, 1%, 2%) of E7-12:OAc and Z7-12:OAc added to 100% Z9-14:OAc, we found that corn-strain males from Peru were not attracted to traps baited only with 2% E7-12:OAc added to Z9-14:OAc, but were equally attracted to all other blends tested (Figure 4). In North Carolina, *S. frugiperda* males were equally attracted to synthetic blends to which E7-12:OAc, Z7-12:OAc or E- and Z-7-12:OAc was added (Figure 4). Unfortunately, we were not able to identify the strain-type of any of the trapped males in North Carolina because of DNA degradation of the samples.

## Discussion

We investigated the variation in attraction of *S. frugiperda* corn- and rice-strain males in North America, the Caribbean and South America, and found a) geographic variation in corn-strain male attraction to synthetic 4-component blends and to different doses of Z7-12:OAc, b) some geographic variation in rice-strain male attraction to different synthetic blends, c) no variation in male attraction to the minor compound Z11-16:OAc, and d) some evidence of geographic variation in response to E7-12:OAc, the compound identified in females from Brazil. Taken together our results indicate that both strains exhibit rather geographic than strain-specific variation in their response to synthetic pheromone lures.

We realize that our field data should be interpreted with some degree of caution for several reasons. Firstly, the composition of the lures of the first experiment (Blend 1 vs. Blend 2) was based on the relative amount of compounds observed in pheromone gland extracts [50], and not from airborne collections, which are somewhat different in composition [58]. We decided to load rubber septa with compound ratios and concentrations that occur in the female gland, assuming a similar emission of volatiles from the pheromone septum as from the female gland surface. Secondly, abiotic conditions may affect the rates at which the pheromone components are released from the lures, as well as the responses of males to a pheromone source [69]. As the experiments were carried out over a large geographic range, it is inevitable that there were intra- and inter-site variability in climatic conditions during the trapping periods. Thirdly, due to the fact that we used mitochondrial markers for strain diagnosis, which are maternally inherited, we were not able to differentiate between homozygote and heterozygote corn- and rice-strain males in our experiments. Therefore, it is possible that also hybrid males were attracted to our lures. Hybridization in the field has been found to be around 16% [70], and several studies have found a bias of inter-strain matings between rice-strain females and corn-strain males, while corn-strain females hybridize less frequently [70]–[73]. Consequently, the majority of hybrids occurring in nature have a rice-strain mitochondrial COI gene. If our lures attracted hybrid males, they were thus probably typed as rice-strain males. However, despite these limitations we believe our findings support the idea of geographic variability in the sexual communication system of the fall armyworm.

### Geographic Variation in Corn-strain Male Responses

Testing two different 4-component blends revealed that corn-strain males were equally attracted to both blends in Canada and North Carolina, but preferred Blend 1 over Blend 2 in South America, i.e. in Argentina and Peru. Interestingly, Blend 1 mimics the pheromone composition previously reported for corn-strain females in Florida [50]. However, corn-strain males in Florida and Puerto Rico showed a preference for Blend 1 in one of the two fields tested at each site, but were equally attracted to Blend 1 and 2 in the other field (Figure 1). This differential male attraction between fields could be caused by habitat-specific volatile differences. For example, the two corn fields in Puerto Rico, which were only 4 km apart, were planted with different corn varieties, were in different phenological states during the trapping period, and were treated with different insecticides. This could result in different background odor profiles, which in turn may have influenced the attraction of corn-strain males to the two different 4-component blends used in the first experiment. Previously, it was shown that corn-strain males varied in their attraction to sex pheromone blends in different fields with different host plants in north and south Florida [52], [55]. Furthermore, both strains have some host plant preferences [42], [46], [47], and *S. frugiperda* males show EAG responses to at least 16 different plant volatiles [74]. Thus, it seems likely that corn-strain males exhibit different responses to female sex pheromones in different habitats, emitting host or non-host volatiles.

The differential attraction of corn-strain males could also be explained by genetic differences between *S. frugiperda* populations from North America, the Caribbean and South America. Population genetic analyses of *S. frugiperda* samples collected throughout the Western Hemisphere generally found no isolation by distance between populations from different regions [75]–[77], which indicates no geographically restricted gene flow probably due to the high migratory ability of *S. frugiperda* [78], [79]. However, these analyses did not take into account strain-specific differences and in several cases the strain-type of captured individuals was unknown [75], [77]. Genetic studies on populations from Arkansas and Florida showed significant genetic variation among populations, both within and between the two strains [80]. Furthermore, corn-strain individuals exhibit different mitochondrial haplotype profiles between populations from a) Florida, Puerto Rico, Georgia and b) Texas, Brazil, Mississippi, Alabama, Louisiana [53], [81]–[83]. Thus, genetic differences could play a role in differential attraction of corn-strain males to synthetic blends in different regions.

Besides different host plant volatiles or genetic differences, the responses of corn-strain males could have been influenced by geographically varying environmental factors like temperature or humidity, which are known to influence sexual communication in insects [69]. In a previous wind tunnel study, we observed that males of both strains were highly sensitive to changes in temperature or humidity and stopped their response to calling females whenever humidity or temperature was low [52]. Thus, it is possible that males may respond differently to pheromone blends in regions with dry and cold climate compared to (sub) tropical climate zones.

In addition to variation in response to two synthetic 4-component blends, corn-strain males also exhibited significant geographic differences in their attraction to different doses of Z7-12:OAc. In Florida and Puerto Rico, corn-strain males were more attracted to the 2% dose than to other doses tested, but were equally attracted to 2% and 4% Z7-12:OAc in Peru (Figure 2). Furthermore, some corn-strain males from Puerto Rico were attracted to Z9-14:OAc alone, even though Z7-12:OAc has been considered an essential secondary sex pheromone component, without which males are not attracted [52],[58]. The fact that the response of corn-strain males to Z7-12:OAc varied between regions suggests that females may also vary in their relative amount of Z7-12:OAc across different regions. Although previous data indicated that the production of Z7-12:OAc is under strong stabilizing selection [52], in light of the data presented here, selection pressures may be different in different regions.

### Geographic Variation in Rice-strain Male Responses

In general, rice-strain males were equally attracted to Blend 1 and Blend 2 in different fields in North America, the Caribbean and South America (Figure 1). Although the response of rice-strain males was significantly different to the one of corn-strain males in a corn field in Puerto Rico and Argentina, the attraction of both strains looks relatively similar in the first experiment. The results of our statistical analysis should be handled with care, as factors like time-dependent density changes within a population, differences between replicates, as well as seasonal/regional differences caused a considerable variation in trap catches (Figure S1). Overall, the results of our first experiment show that both strains respond similarly to the two different 4-components blends that we tested, but exhibit some geographic variation in their response.

Interestingly, rice-strain males from Florida and Puerto Rico showed a broader response spectrum to different doses of Z7-12:OAc compared to corn-strain males (Figure 2). This small but significant strain-specific difference is likely to be important, because Z7-12:OAc is the critical secondary sex pheromone component which is usually crucial for male attraction [52], [58]. However, it is possible that the broader response spectrum of rice-strain males is due to attraction of hybrid males, if hybrids prefer more extreme blend ratios than the parental pure strains. This should be tested further, although we do not assume that the response of rice-strain males is masked by the presence of hybrid males, because hybridization frequency in nature is relatively low [70]. In conclusion, the results of our experiments suggest that both strains exhibit rather geographic than strain-specific differences in their response, although the response of rice-strain males seems to be broader than that of corn-strain males.

### Male Attraction to the Minor Compound Z11-16:OAc

Testing the importance of Z11-16:OAc for male attraction showed that corn-strain males from Peru were equally attracted to blends with and without different doses of Z11-16:OAc, similar to the response of corn- and rice-strain males in Florida [52]. In general, addition of Z11-16:OAc to Z9-14:OAc and Z7-12:OAc did also not decrease male attraction in Florida, Peru, Costa Rica and Pennsylvania [52], [58], [61], [62]. These data suggest that Z11-16:OAc is not an essential component for *S. frugiperda* male attraction, which is supported by the observation that *S. frugiperda* males from Mexico did not respond electrophysiologically to Z11-16:OAc [74].

### Geographic Variation in Male Attraction to E7-12:OAc

So far, the E-isomer of the critical secondary sex pheromone component Z7-12:OAc has only been found in *S. frugiperda* females from Brazil, and males from this region responded electrophysiologically to E7-12:OAc and exhibited a higher attraction to binary blends (Z9-14:OAc and Z7-12:OAc) when E7-12:OAc was added [59]. In our trapping experiments, we found that corn-strain males from Peru were not attracted to traps baited only with E7-12:OAc and Z9-14:OAc, but were similarly attracted to all other blends that contained Z7-12:OAc (Figure 4). Thus, corn-strain males from Peru appear to distinguish between both isomers and need Z7-12:OAc, but not E7-12:OAc, for attraction. This result contrasts our findings in North Carolina, where males did not differentiate between these two isomers. However, while in Peru males captured were corn-strain individuals, males caught in North Carolina could not be strain-typed due to DNA degradation, but probably belonged to both strains. Hence, we currently cannot exclude the possibility that corn- and rice-strain males show differential strain-specific attraction to E- and Z-7-12:OAc. Different isomers of a pheromone component are usually critical for attraction of males and can even lead to speciation, as shown in the two pheromone strains of *Ostrinia nubilalis* (Hübner) [84]. Taken together, geographic variation in response to E7-12:OAc seems to exist, but additional experiments are required to evaluate the importance of E7-12:OAc for both strains in different regions.

### Implication for Pest Management

As the fall armyworm is a serious agricultural pest, an efficient monitoring system for both strains in different habitats and regions would be helpful to detect infestations early and start pest control. To avoid high costs, monitoring via pheromone baited traps requires an effective “minimal” synthetic lure that equally attracts both strains in all habitats and regions. The results of our study showed that only Z9-14:OAc and Z7-12:OAc are usually required to attract *S. frugiperda* in the field, and although both strain exhibit some strain-specific responses towards different doses of Z7-12:OAc, their response ranges also overlap (Figure 2). More precisely, both strains responded equally well to 2% Z7-12:OAc and therefore, we recommend a monitoring blend consisting of 100% Z9-14:OAc (300 µg) and 2% Z7-12:OAc (6 µg).

### Conclusions

Corn- and rice-strain males exhibited some similarities in their attraction to the different blends that we tested, although corn-strain males showed more differentiation in their response than rice-strain males. We found some geographic variation in attraction of corn- and rice-strain males to two synthetic 4- component blends. In contrast, rice-strain males, but not corn-strain males, showed almost no geographic variation in their attraction to different doses of Z7-12:OAc in different regions. One aspect that merits further attention is the possibility that habitat-specific volatiles influence the male response to pheromone blends in different fields. Furthermore, the minor compound Z11-16:OAc does not seem to affect attraction of *S. frugiperda* males, while region-specific differences in the attraction seem to occur to the compound that has only been identified from female glands in Brazil, E7-12:OAc. Overall, the data show some geographic variation in the response of *S. frugiperda* males to pheromone blends. If this variation coincides with geographic variation in the female pheromone composition, then geographic differentiation between populations could occur.

## Supporting Information

Figure S1 **Variation in trap catches of** ***Spodoptera frugiperda*** **males in different fields and regions.** Graphs show the total number of corn- and rice-strain males that were caught with Blend 1 (100% Z9-14:OAc +13% Z11-16:OAc +2% Z7-12:OAc +1% Z9-12:OAc) in different regions in the first experiment (Figure 1). All traps were rotated three times and all replicates were conducted in the same field, except for North Carolina, where each replicate was conducted in a different field (replicate 1: soybean field, replicate 2: cotton and grass field, replicate 3: soybean and corn field).
(TIF)
