# Supplementary figures and images for: Geographic Variation in Sexual Attraction of Spodoptera frugiperda Corn- and Rice-Strain Males to Pheromone Lures
Source: PLoS One. 2014 Feb 19;9(2):e89255. doi: 10.1371/journal.pone.0089255 (PMC3929749; doi:10.1371/journal.pone.0089255)

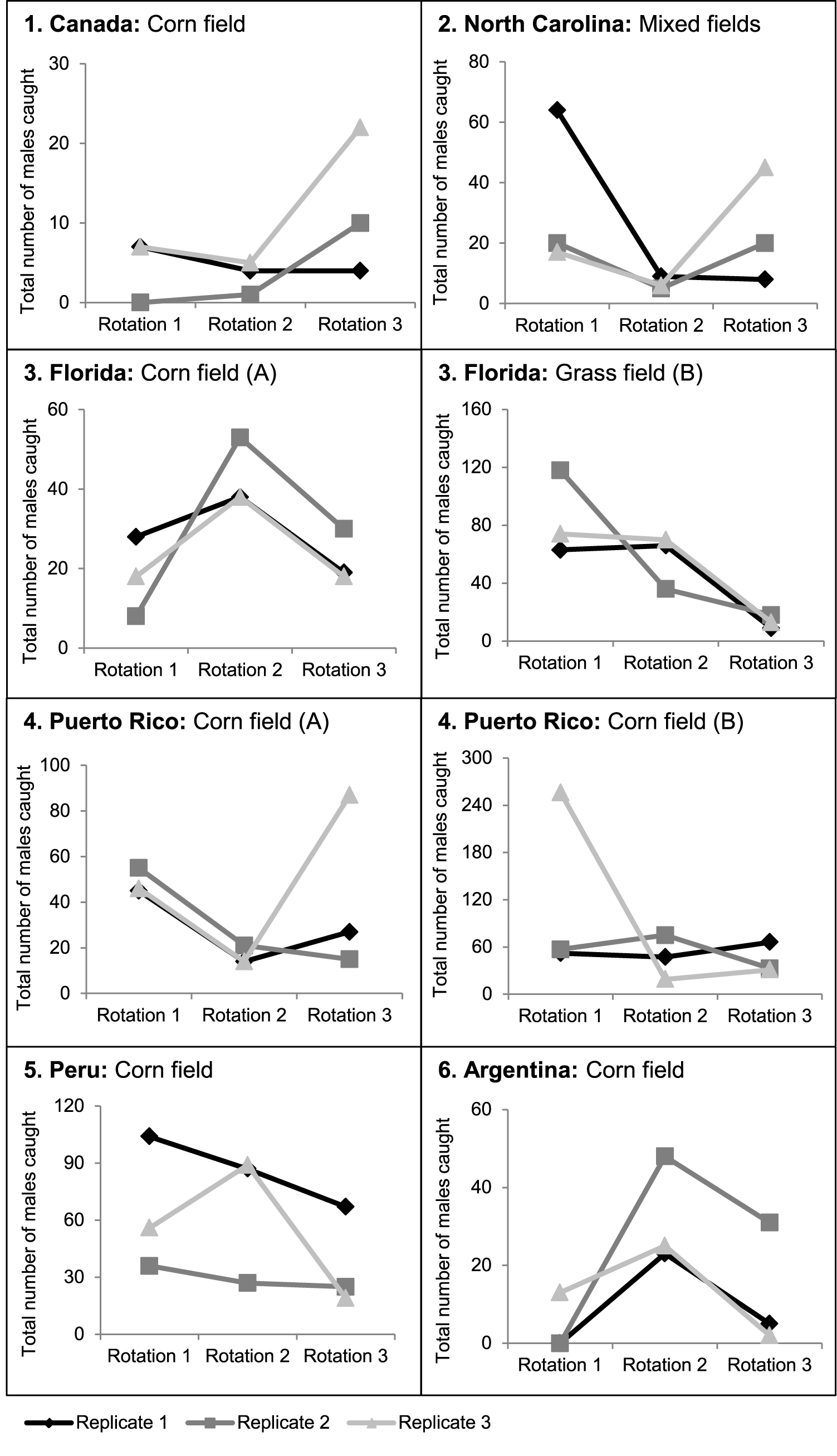

Supplement: Figure S1 — Variation in trap catches of Spodoptera frugiperda males in different fields and regions. Graphs show the total number of corn- and rice-strain males that were caught with Blend 1 (100% Z9-14:OAc +13% Z11-16:OAc +2% Z7-12:OAc +1% Z9-12:OAc) in different regions in the first experiment (Figure 1). All traps were rotated three times and all replicates were conducted in the same field, except for North Carolina, where each replicate was conducted in a different field (replicate 1: soybean field, replicate 2: cotton and grass field, replicate 3: soybean and corn field). (TIF) [file pone.0089255.s001.tif]
